# The Efficacy of Concentrated Growth Factor in the Healing of Alveolar Osteitis: A Clinical Study

**DOI:** 10.1155/2020/9038629

**Published:** 2020-05-12

**Authors:** Aqsa Kamal, Basheer Salman, Noor Hayati Abdul Razak, Ali Al Qabbani, A. R. Samsudin

**Affiliations:** ^1^College of Dental Medicine, University of Sharjah, Sharjah, UAE; ^2^School of Dental Sciences, Universiti Sains Malaysia, Penang, Malaysia

## Abstract

**Background:**

A dry socket also referred to as alveolar osteitis (AO) is a common postoperative complication following tooth extraction, due to the disruption of the clot within the wound. This study aimed to evaluate the efficacy of concentrated growth factor (CGF) in the healing of alveolar osteitis following tooth extraction.

**Methods:**

The study was conducted at University Dental Hospital Sharjah, UAE. Patients undergoing tooth extraction at the oral surgery clinic were advised to return immediately if they suffer from pain. Over the following first week after tooth extraction, patients who reported pain symptoms were recalled and all dry sockets were identified. The patients were divided into two groups. Group I patients received conventional treatment with socket curettage and saline irrigation only, while in group II CGF was inserted into the socket. Both groups were observed for pain score and quantification of granulation tissue formation.

**Results:**

A total of 40 dry socket patients, aged between 18 and 60 years, from a total of 1,250 patients, were included in the study. 30 patients were given conventional treatment while another 10 patients were given CGF. Patients who received CGF had a pain score of 7–10 at presentation, and the pain score dropped to 0–3 on day 4 and further improved to 0-1 on day 7 (*p* = 0.001). Granulation tissue formation appeared in the conventional group I on day 7 while the CGF group II showed earlier granulation tissue formation by day 4 (*p* = 0.001). The posttreatment pain score is inversely proportional to the amount and rate of granulation tissue formation in the socket.

**Conclusion:**

The study suggests that delivery of CGF into a dry socket helps relieve pain and expedite the wound healing process as shown by a statistically much lower pain score and earlier and more rapid formation of granulation tissue when compared to the conventional alveolar osteitis therapy.

## 1. Introduction

A dry socket also referred to as alveolar osteitis is a postoperative complication following tooth extraction. Both terms “dry socket” and “alveolar osteitis” have been used interchangeably in the dental literature. It can be defined as “postoperative pain within and around the extraction site, which rises in severity at any time between the first and fifth days after the tooth extraction, accompanied by a partial or complete disintegration blood clot within the alveolar socket and with or without halitosis” [1]. It occurs in 0.5–5% of routine dental extractions and 25–30% following the extraction of impacted mandibular wisdom tooth [2, 3]. Females are more frequently affected than males, but this appears to be related to oral contraceptive use rather than any underlying gender predilection [4]. Unlike other forms of wound infection, alveolar osteitis occurs frequently in the young age group, although wound infection, in general, is more likely to occur with increasing age.

Even, in this era of cell and molecular biology, the specific etiology of the dry socket has not yet been defined. However, numerous local and systemic elements make contributions towards it [1, 5, 6]. Some of the risk factors are difficult surgical extraction, trauma, microbiological origin, smoking, age, and contraceptive pill use [7, 8]. Presence of periodontal disease, acute necrotizing ulcerative gingivitis, local bone disease, or previous history of developing a dry socket has also been implicated [3, 9, 10].

**Figure 1:**
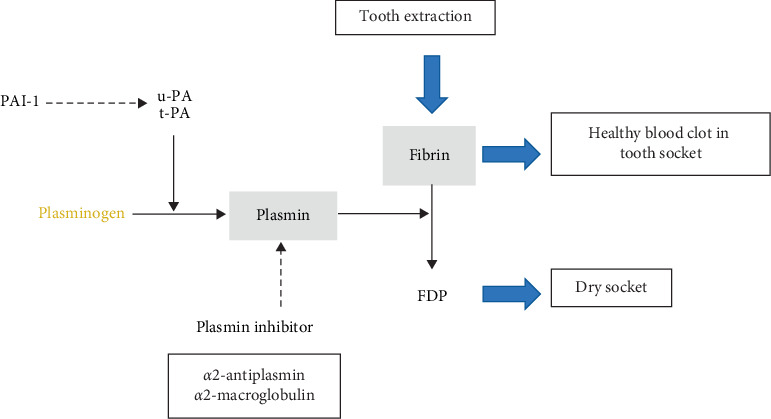
Molecular mechanism of alveolar osteitis. Plasminogen type-1 is the precursor of plasmin which acts in fibrin degradation. Plasmin inhibitor sterically shields the active site of plasmin, thus substantially decreasing plasmin's access to protein substrates [15]. t-PA: tissue-type plasminogen activator; FDP: fibrin degradation products.

Clinical and experimental research studies have described an elevated fibrinolytic activity as a major factor for the etiology of the dry socket [6, 11–14]. Human plasminogen is a single-chain glycoprotein containing 791 amino acid residues and 2% carbohydrate. In these studies, plasminogen type-1, released following a routine tooth extraction, is the precursor of plasmin, a potent serine protease involved in the dissolution of fibrin clots (Figure 1). Type-1 plasminogen appears more readily recruited to blood clots [6, 16]. Tissue-type plasminogen activator (t-PA) is the principal endogenous activator of plasminogen in blood. It is produced as a single-chain molecule by the vascular endothelial cells and is secreted into the plasma by an acute release following stimulation of certain endothelial cell receptors such as in tooth extraction injury. It is also known that plasmin deficiency may lead to thrombosis, as clots are not adequately degraded. Plasmin activity is inhibited mainly by binding to the plasmin inhibitor, which forms a stable complex with plasmin devoid of proteolytic activity [6, 16].

Being an old surgical puzzle, many different methods have been advocated to treat alveolar osteitis, such as the application of local turmeric [17], zinc oxide eugenol, alvogyl, honey or vitamin C [18], and socket irrigation with hydrogen peroxide [19]. However, these conventional treatment approaches merely solve the symptoms but do not target the key etiology. Furthermore, most of these approaches do not completely alleviate the pain and other symptoms. More recent approaches come into play based on experience of wound healing management in other surgical specialty areas, and these include the use of low intensity pulsed ultrasound therapy (LIPUS) [20], low level laser therapy (LLLT) [21, 22], ozone therapy [23, 24], and the use of platelet rich plasma (PRP) in general and oral wound healing.

As the knowledge on the biology of wound healing advances, the role of cytokines and growth factors in the healing of alveolar osteitis becomes more significant [25]. Understanding the molecular aspects of wound healing plays an important role in dry socket healing. Over the past two decades, platelet rich plasma (PRP) has been used in many surgical fields as an additional remedy for supporting wound healing. Platelet concentrates are attractive autologous scaffolds suitable for regenerative medicine with its fiber architecture and rich growth factors [26]. Concentrated growth factor (CGF) is the third generation of autologous plasma extract prepared by a special centrifugal program [27]. Its application in the oral region is still controversial particularly when oral wound healing shows different immunologic responses and regenerative capabilities compared to general surgical wounds [28, 29]. Therefore, the aim of this study is to investigate the efficacy of CGF as a local treatment option for alveolar osteitis (AO) compared with conventional therapy.

## 2. Materials and Methods

This study was conducted from August till October 2019 at University Dental Hospital Sharjah (UDHS), United Arab Emirates. Human ethics approval was obtained from the Research Ethics Committee University of Sharjah REC-17-02-14-01-S dated 24^th^ October, 2017. The study population was patient treatment at the oral surgery clinic of University Dental Hospital Sharjah. The subjects included were patients who underwent routine tooth extractions performed under local anesthesia following the diagnosis of irreversible pulpitis, chronic and acute apical pathology, periapical abscesses, and advance periodontitis. Other reasons for tooth extractions and the presence of local odontogenic tumors or malignancies were excluded. Patients with well-controlled systemic medical conditions such as diabetes and hypertension were included in the study. Following tooth extraction, the socket was curetted in cases of periapical pathology and patients were asked to bite on a piece of sterile gauze for 30 minutes to achieve hemostasis. There were no additional dressings or medication inserted in the socket. The presence of a nice healthy clot as observed by the attending dentist was confirmed before the patients were discharged from the clinic. They were advised to return immediately to the University Dental Hospital if they suffer from postoperative pain. Complicated tooth extractions under local anesthesia that lead to minor oral surgical procedures were excluded. All patients included in the study were not prescribed antibiotics. Over the following five days following the tooth extraction, the patients who reported suffering from postoperative pain symptoms were reexamined in the clinic.

**Figure 2:**
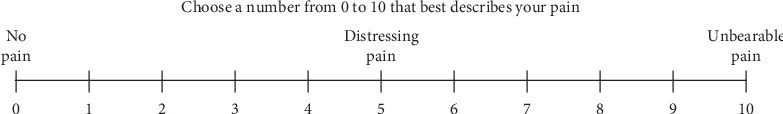
Visual analogue pain scale (VAS) for recording the pain level of patients for dry socket [30, 31].

Patients who were diagnosed with alveolar osteitis were referred to the oral surgery department. The selection of the subjects was done according to the previous inclusion and exclusion criteria. All patients aged between 18 and 60 years who were diagnosed with alveolar osteitis were included in the study. Those with good oral hygiene who had undergone nonsurgical tooth extraction under local anesthesia and who suffered postoperative pain between 1 and 5 postextraction days presenting with empty dry socket were selected. Patients aged below 18 and above 60 years and those who had tooth extraction performed outside UDHS or patients who underwent surgical extraction were excluded. The patient's pain level was recorded on a visual analogue scale of 1 to 10 [30, 31] (Figure 2), and their body temperature was also recorded.

The selected patients were divided into two groups based on their choice to be treated with either conventional treatment (group I) or CGF treatment (group II).

In group I the patients were treated with conventional treatment whereby the dry socket was curetted and irrigated with saline under local anesthesia. A new bleeding socket was created following curettage and gentle saline irrigations help debride the necrotic debris. The patient was instructed to bite on a piece of sterile gauge to achieve hemostasis.

In group II, the socket was similarly curetted and irrigated under local anesthesia and CGF in gel form was inserted into it. Preparation of CGF was performed by obtaining about 9 ml of the patient's blood into a vacuum test tube. CGF was prepared using Medifuge centrifuge machine, Silfradent, Italy, following a cycle duration of 5 minutes at 1,000 revolutions. The processing time is about 12 minutes and finally a thick yellowish color gel layer was produced known as CGF. This CGF gel was directly delivered into the socket using a surgical tweezer [32–34].

A periapical radiograph of the socket was done for both groups before initiating treatment to exclude the presence of retained apices, bony fragments, and fracture of the alveolus. No dressing such as alvogyl or topical antibiotic was placed in both groups and no systemic antibiotic was prescribed.

The day of the patient's return to the clinic with a dry socket presentation was recorded as “Day 0.” Both groups were observed for pain score using a visual analogue pain scale from 1 to 10 (Figure 2) and quantification of clinically evident granulation tissue formation within the socket was recorded on day 0, day 4, and day 7.

Continued follow-up of patients in both groups was done through phone calls on a weekly interval for up to 4 weeks after dry socket treatment.

### 2.1. Statistical Analysis

All the data was entered into SPSS software. We used Kruskal–Wallis test to determine the mean and standard deviation for age and scoring of pain and granulation tissue formation.

A *p* value of less than 0.05 is considered significant.

## 3. Results

Over a three-month period, a total of 40 dry socket patients, 25 males and 15 females, were included in the study (see Table 1) from a total of 1,250 patients who attended for routine tooth extraction under local anesthesia at oral surgery clinic, University Dental Hospital Sharjah. Among the dry socket cases, five patients were noted to be diabetic, four patients were hypertensive, three patients were both diabetic and hypertensive, and one patient had other medical conditions. Only three male patients were smokers and their ages were between 35 and 49 years. This study showed that more patients accepted the conventional treatment compared to the new CGF treatment. In group I, 30 patients aged between 18 and 60 were given conventional treatment while another 10 patients in group II aged between 18 and 60 were given CGF.

In the conventional group I, the pain score was 7–10 on the day of presentation (day 0) and the pain score dropped to 4–6 on day 4 and further decreased to 2–4 on day 7 (see Table 2) following treatment. Granulation tissue (GT) formation within the healing socket in group I appeared clearly only on day 7. In group II patients who received CGF, a similar pain score of 7–10 was recorded on the day of presentation (day 0), and the pain score dropped to 0–3 on day 4 and further improved to 0–1 on day 7 (see Table 2).

Quantification of clinically evident granulation tissue formation within the socket was recorded (see Table 3), from day 0, day 4, and day 7 after dry socket treatment. A completely barren dry socket without granulation tissue GT was recorded as (nil). The formation of GT in one quarter or less of the socket was recorded as (+), while formation of granulation tissue in half of the socket was recorded as (++), formation of granulation tissue in three quarters was recorded as (+++), and complete laying down of granulation tissue within the whole socket was recorded as (++++).

The CGF applied sockets among group II patients also showed much earlier granulation tissue GT by day 4. By the second week postoperatively, both groups showed similar pain score and GT formation and showed comparable wound healing stage after 14 days of post dry socket treatment. The results were tested statistically (see Tables 4 and 5) to see the significance.

## 4. Discussion

In this study, the incidence of the dry socket in University Dental Hospital Sharjah is around 3%. The incidence of dry socket has been reported as 1–4% world wide following routine dental extractions, with the incidence increasing to 10 times greater for lower as compared to upper teeth, and reaching 45% for mandibular third molar removal [1, 35]. Our data is quite consistent with many other studies and it is interesting to note that dry socket incidence does not seem to run parallel with clinical standards of infection control since our infection control protocol is much higher than many other centers that report the same incidence. This fact may suggest a controversy against the microbial etiology for dry sockets.

There are more dry socket subjects within the middle age group among the 40 patients seen at University Dental Hospital Sharjah. The mean age of dry socket subjects was 38.03 years. More male patients were seen with dry sockets although the University Dental Hospital records show almost an equal number of both male and female patients undergoing tooth extraction on a daily basis. Interestingly, male was predominantly ranging from 30 to 49 years and less females were seen, mostly in the age range from 30 to 49 years. The lesser number of females may be due to lower contraceptive drug consumption in the United Arab Emirates [36], since more than 50% of women in childbearing age do not favour contraception, and this factor may contribute to a lower number of alveolar osteitis among women [36]. Use of oral contraceptives has been shown to significantly increase the risk of dry socket since estrogens and other risk factors such as surgical trauma are thought to cause dry socket by stimulating fibrinolysis [37, 38].

Incidence of alveolar osteitis commonly occurs among patients aged 18 years or older as compared to younger individuals [4, 6]. Dry socket rarely occurs in children and some other etiology must be suspected if it happens in the pediatric age group [39]. Some studies suggest that alveolar osteitis rarely occurs during childhood, because this is a period in which micro-organisms such as *Treponema denticola* are usually not detected in the oral environment, and such bacteria have not colonized in the pediatric oral cavity [8]. Young adults and middle-aged individuals are more prone to dry sockets due to a much higher immunological response to injury compared to the older age group. Some studies suggest that age has an inverse relation with the formation of alveolar osteitis [35] which is in contrast to our findings since there were very few patients who were in 18–29 years of the age range that suffer from alveolar osteitis.

We noted that all patients suffer severe pain with a VAS pain score of 7–10 on the day of presentation. The lysis of the blood clot in the socket leads to fibrin degradation products attributed to the formation of kinins in the compact socket. Plasmin is involved in the conversion of kallikrein into kinins and together with other inflammatory mediators causes intense pain. Following conventional treatment under local anesthesia, the pain score dropped to VAS 4–6 on day 4 and clinical findings showed some laying down of GT formation within the socket. In the sockets treated with CGF, a much more drastic improvement in pain symptom was shown with a VAS of 0–3 as early as day 4, and clinical evaluation showed a much richer pool of granulation tissue within the healing socket. This observation demonstrated that the amount and rate of granulation tissue (GT) formation in the socket are inversely proportional to the pain score and there is a significant effectiveness of CGF on the healing of dry socket and improvement in symptoms (*p*=0.001).

None of the patients in both groups showed a rise in body temperature. This shows that alveolar osteitis is a local condition without systemic upset. This study captured a few dry socket patients with controlled diabetes and hypertension. All patients included in this study were in good health. Most studies suggest that systemic conditions play a vital role in the etiology of alveolar osteitis [40]. However, many authors do agree that both healthy and medically compromised patients with systemic conditions such as being diabetic may succumb to dry socket following normal tooth extraction. The question of whether medically compromised patients are more prone to suffer from dry sockets is difficult to quantify. Furthermore, our University Dental Hospital is a referral center for medically compromised dental patients but there were very few medically compromised patients who underwent tooth extraction and succumbed to a dry socket complication. It must be emphasized that only well-controlled diabetics and hypertensive patients are permitted to undergo elective tooth extractions at the University Dental Hospital Sharjah. Therefore, this study suggests that patients with well-controlled systemic diseases are good candidates for tooth extraction and there are yet other unknown factors that compromise early healing of the socket resulting in alveolar osteitis.

Smoking is known to interfere with wound healing. Only three smokers suffered from dry sockets in this study. There are different studies which suggest that smoking is a strong predisposing factor for the formation of the dry socket [41], although most subjects in this study deny current smoking. We did not take the past history of cigarette smoking since it should be noted that cigarette smoking is an expensive habit in UAE. It is reported that self-reported tobacco use in UAE is 36% among men and 3% in women [42]. Smoking as a strong etiology also makes the incidence of dry sockets be lower among children and females too.

In the conventional treatment approach, local anesthesia is given to patients followed by curettage of the bare necrotic bony socket to induce bleeding, and the socket was irrigated with normal saline. This is done to induce clot formation, by subjecting the wound again through all the phases of normal wound healing beginning from hemostasis, acute inflammation, proliferation, maturation, and remodeling. Platelets aggregates in severed vessels trigger the clotting cascade and release essential growth factors and cytokines that are important for the initiation and progression of wound healing. The fibrin matrix resulting from this stabilizes the wound and provides a provisional scaffold for the wound healing process. Curettage of the socket followed by irrigation with physiological saline allows debridement of necrotic soft tissue and bone debris supporting the development of a vital base for the invasion of new capillaries, laying down of fibroblast matrix, and granulation tissue formation.

Curettage of the dry socket was performed in both groups of patients. However, there are advocators who do not favour socket curettage for fear of inflicting further trauma [43]. Failure of adequate socket debridement will not favour the early invasion of fibrovascular tissue into the poorly vascularized wound and dependence on natural phagocytosis will take an extremely lengthy healing period.

Treatment with CGF showed a higher potency of the healing process. Concentrated growth factors are ideal for clotting as it contains essential growth factor: platelet-derived growth factor (PDGF), transforming growth factor (TGF), platelet factor interleukin (IL), vascular endothelial growth factor (VEGF), epidermal growth factor (EGF), insulin-like growth factor IGF, and fibronectin [34]. Together, this cocktail of growth factors speeds up the development of the delicate fibrovascular granulation tissue. Different studies have shown that the presence of growth factors can speed up the healing process [44, 45]. CGF promotes cell proliferation and migration and regulates the biological behavior of diverse cell types and supports angiogenesis which is a key element in any wound healing process [27].

In this study, patients were advised to come back immediately in case of any pain following the tooth extraction. Most patients reported having pain after 4–7 days. However, all patients started to have pain from the very next day after the tooth extraction but due to their job circumstances, no leave from work, family commitments, and other factors they tend to return to the clinic after few days. It was observed that acute pain develops almost immediately following lysis of the clot and exposure of the unprotected dry bony socket and nerve endings, with the production of kinins and inflammatory mediators. Almost all the patients had a pain score from 7 to 10 on the visual analogue pain scale associated with tenderness of the regional lymph nodes. The severe pain from the dry socket is often underestimated by the attending dentist.

An interesting observation in this study is the absence of pus discharge from the socket and afebrile status of a patient's body temperature. Many dental practitioners may misinterpret the yellowish necrotic bone slough as pus discharge from the socket. The absence of a rise in body temperature also signifies a dry socket to be a local wound dehiscence event without systemic physiological upset. The conventional treatment approach for the dry socket helps alleviate the pain through the formation of granulation tissue but recovery was slow. This phenomenon suggests that reformation of the natural blood clot (or some may call them secondary clot) in the treated dry socket is not as effective as the first natural clot formed immediately after tooth extraction. The addition of CGF will enhance the compromised weaker secondary clot. CGF gel is known to have closely interwoven fibers exhibiting a relatively stiffer texture than the traditional platelet rich fibrin (PRF) and able to promote cell adhesion and migration [46] during the proliferative healing phase.

Rutkowski et al., in 2010, used PRP to promote bone formation following extraction [33]. They observed the positive effects of PRP on bone formation and wound healing. Haraji et al., in 2012, also worked on dry socket treatment. They treated patients with PRP and showed that their beneficial properties are very effective in the treatment of dry sockets and in normal healthy extraction sockets including bone healing [32]. Chenchev et. al., in 2017, worked on alveolar osteitis using platelet rich fibrin (PRF). They too claimed that PRF can successfully treat dry socket by reducing pain symptoms and expedite wound closure and epithelialization [15, 45]. King et al. [47] worked on PRP as plasma rich in growth factors, to treat alveolar osteitis in 2018. They claimed that plasma rich in growth factors indeed has considerable advantages [47].

Granulation tissue is young connective tissue possessing microscopic blood vessels which is formed on the surface of a wound during the healing process, forming a protective layer, and resistant to microbial invasion. It is friable and may be easily damaged, but CGF seems to promote the formation and protection of granulation tissue in a compromised wound. This could be the main reason for the dry socket healing expedition and reduction of pain.

Our study showed that conventional dry socket treatment does provide palliation but the addition of CGF expedites the healing process and alleviates the symptoms much earlier [34]. It further demonstrates that conventionally treated sockets take more than 7 days to match the effective healing of a CGF treated socket. CGF works at the molecular level, targeting some of the key physiological deficiencies of the dry socket [32–34]. The use of autologous platelet concentrates may be helpful because of their local immunomodulatory properties and possible promotion of angiogenesis and tissue healing by platelet factors [48]. CGF, like other second-generation autologous platelet concentrates, is easy to prepare, is of low cost, and has minimal risk for the patient [49].

## 5. Conclusion

This study suggests that the delivery of CGF into a dry socket helps to relieve pain and expedite the wound healing process as shown by a much lower pain score and earlier and more rapid formation of granulation tissue when compared to conventional therapy. Chairside CGF techniques in the dental office are simple, feasible, and economical with predictable results.

### 5.1. Observed Learning Points

- Pain from the dry socket is excruciating and often underestimated by the attending dentist
- Dry socket is a localized failure of tooth extraction wound healing
- It causes neither a systemic upset nor a rise in body temperature
- Low contraceptive consumption in the community tends to produce a lower prevalence of dry sockets among women in childbearing age
- Dry socket does not occur in children
- Concentrated growth factor supports dry socket healing by expediting granulation tissue formation and alleviating pain

## Figures and Tables

**Table 1:** Patient demography.

	Mean (standard deviation, SD)	Frequency (percentage %)
Age	38.03 (8.96)	
Gender		
Male		25 (62.5)
Female		15 (37.5)

**Table 2:** Pain and granulation tissue (GT) score of subjects in group I and group II at days 0, 4, and 7.

Patient no.	Age	Gender	Treatment option	Day 0		Day 4		Day 7	
				Pain before treatment	GT	Pain	GT	Pain	GT
1	53	Female	CGF	7	Nil	0	+	0	++
2	39	Female	CGF	8	Nil	0	++	0	+++
3	28	Male	Control	10	Nil	6	Nil	3	+
4	35	Male	Control	9	Nil	5	+	5	+
5	38	Male	Control	8	Nil	6	Nil	3	+
6	33	Male	CGF	9	Nil	1	++	0	+++
7	38	Male	CGF	8	Nil	0	++	0	++
8	29	Male	CGF	9	Nil	0	+	0	+++
9	40	Female	Control	10	Nil	5	Nil	3	+
10	49	Female	Control	8	Nil	4	Nil	3	+
11	42	Male	Control	9	Nil	5	Nil	0	+
12	40	Female	CGF	9	Nil	1	++	0	+++
13	38	Female	CGF	7	Nil	0	+	0	+++
14	40	Male	CGF	7	Nil	0	++	0	++
15	35	Male	Control	8	Nil	4	Nil	0	+
16	32	Female	Control	8	Nil	5	Nil	4	+
17	49	Male	CGF	8	Nil	1	++	0	++
18	31	Male	CGF	8	Nil	0	++	0	+++
19	42	Female	Control	7	Nil	3	Nil	5	+
20	36	Male	Control	8	Nil	5	Nil	3	+
21	27	Male	Control	10	Nil	6	Nil	4	+
22	42	Female	Control	8	Nil	5	+	3	+
23	36	Male	Control	9	Nil	6	Nil	4	+
24	51	Male	Control	8	Nil	4	Nil	3	+
25	35	Female	Control	10	Nil	6	+	3	+
26	49	Male	Control	9	Nil	7	Nil	2	+
27	60	Male	Control	9	Nil	6	Nil	3	+
28	40	Female	Control	8	Nil	5	Nil	3	+
29	47	Male	Control	8	Nil	7	Nil	2	+
30	40	Female	Control	7	Nil	7	Nil	4	+
31	57	Male	Control	10	Nil	6	+	4	++
32	36	Female	Control	8	Nil	6	Nil	4	+
33	23	Male	Control	9	Nil	6	+	4	++
34	23	Male	Control	8	Nil	5	Nil	4	+
35	27	Male	Control	8	Nil	4	Nil	3	++
36	23	Male	Control	8	Nil	4	Nil	2	+
37	40	Female	Control	10	Nil	4	+	2	++
38	38	Male	Control	10	Nil	5	+	3	++
39	30	Male	Control	9	Nil	4	Nil	3	+
40	30	Female	Control	9	Nil	4	Nil	2	+

**Table 3:** Scoring system employed for granulation tissue (GT) formation in the dry socket.

Clinical presentation of GT	GT presence	GT score
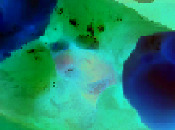	No GT	Nil
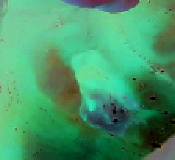	GT in one quarter or less	+
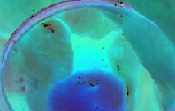	GT in two quarters	++
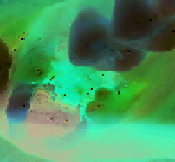	GT in three quarters	+++
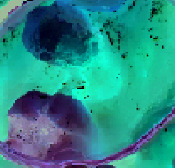	GT in four quarters	++++

**Table 4:** Pain score among groups I and II.

Day	Group	Mean rank	*p* value
Day 0	Group I	22.37	0.065
	Group II	14.90	

Day 4	Group I	25.50	0.001
	Group II	5.50	

Day 7	Group I	25.17	0.001
	Group II	6.50	

**Table 5:** Granulation tissue score in groups I and II.

Day	Group	Mean rank	*p* value
Day 4	Group I	16.08	0.001
	Group II	33.75	

Day 7	Group I	15.83	0.001
	Group II	34.50	
